# Metal stent for the ureteral stricture after surgery and/or radiation treatment for malignancy

**DOI:** 10.1186/s12894-021-00912-6

**Published:** 2021-10-16

**Authors:** Wei Wang, Xiaoshuai Gao, Jixiang Chen, Zhenghuan Liu, Liao Peng, Xin Wei

**Affiliations:** Department of Urology, Institute of Urology, West China Hospital, Sichuan University, No. 37 Guo Xue Xiang, Chengdu, Sichuan 610041 People’s Republic of China

**Keywords:** Metal stent, Ureteral stricture, Iatrogenic

## Abstract

**Background:**

To assess the efficacy and safety of self-expanding metal ureteral stent for the stricture following surgery and/or radiation for malignancy.

**Methods:**

We performed 36 metal ureteral stent insertion procedures (32 patients) between May 2019 and June 2020. The main inclusion criterion was the patients with ureteral stricture due to surgery and/or radiation treatment for malignancy. The diagnosis of stricture was ascertained by history and radiographic imaging. The etiologies underlying the strictures were: surgery and/or radiation therapy for cervical and rectal cancer, surgery for ovarian cancer. The primary outcome was the stent patency rate, and the secondary outcomes were the postoperative complications and glomerular filtration rate (GFR). Stent patency was defined as stent in situ without evident migration, unanticipated stent exchange or recurrent ureteral obstruction. Cost analysis was calculated from stent cost, anesthesia cost and operating room fee.

**Results:**

The pre-metallic stent GFR was 22.53 ± 6.55 mL/min/1.73 m^2^. Eight patients were on double-J stents before insertion of metallic stents. The total annual cost of per patient in our study was $10,600.2 US dollars (range $9394.4–$33,527.4 US dollars). During a median follow-up time of 16 months (range 8–21 months), 27 cases (31 sides, 84%) remained stent patency. Twelve patients died from their primary malignancy carrying a patency stent. Stent migration was observed in 4 patients within 10 months after insertion. Ectopic stents were endoscopically removed and replaced successfully. Three stents were occluded, and no encrustation was seen in our study. Three and four patients had postoperative fever and gross hematuria, respectively. Infection was observed in 2 cases, mandating antibiotics therapy. In addition, postoperative volume of hydronephrosis postoperatively was significantly reduced compared with preoperation (54.18 ± 15.42 vs 23.92 ± 8.3, *P* = 0.019). However, no statistically significant differences regarding GFR, creatinine levels, blood urea nitrogen and hemoglobin existed between preoperation and last follow-up.

**Conclusions:**

The current study demonstrated that metal ureteral stent is effective and safe in the treatment of stricture following surgery and/or radiation therapy for malignant cancer. Patients hydronephrosis could be improved by the stent placement.

## Introduction

It is reported that nearly 75% of all ureteral strictures were caused by Iatrogenic injury and radiation treatment [1]. If left untreated, prolonged obstruction and narrowing of ureter can lead to hydronephrosis, decreased renal function, renal failure and even loss of a renal unit. Timely removal of obstruction is the golden principle for treating ureteral strictures. Currently, there are several therapeutic options available, including open surgery, laparoscopic or robotic reconstruction surgery, endoscopic ureterotomy, nephrostomy and stent insertion. For patients who developed ureteral stricture after surgery and/or radiation treatment for malignancy, it is more difficult to conduct open surgery again due to the formation of intra-abdominal adhesion in this scenario. Nephrostomy and double-J stent insertion are commonly used in these patients, but they are associated with high complication rates and require frequent replacement with increased medical cost [2–4]. Moreover, double-J stent is prone to occlude because of extrinsic compression [5].

Allium® ureteral stent is a self-expanding, large caliber stent. It consists of a super-elastic nickel-titanium alloy to provide long-term direct wall support. One retrospective study had determined the role of Allium stent in the treatment of malignant ureteral stricture [6]. However, they failed to assess the change of hydronephrosis and renal function before and after stent placement procedure, and they did not present cost analysis in their study [6]. Therefore, we presented our experience with the Allium® ureteral stent in the treatment of stricture following surgery and/or radiation treatment for malignancy. We evaluated the stent patency rate and complications during a median follow-up time of 16 months, the hydronephrosis and glomerular filtration rate (GFR) improvement after stent placement was also assessed. Moreover, cost analysis of Allium stent was performed for the first time.

## Method

This prospective clinical trial was approved by the Ethics Approval Committee of West China Hospital, and the registered number was 2019-009. The current study was conducted according to the international ethical recommendations stated in the Declaration of Helsinki and its later amendments or comparable ethical standards [7]. All participants were informed of the risks and benefits of stent placement. The inclusion criterion was that the patients with ureteral stricture due to surgery and/or radiation treatment for malignancy. The diagnosis of stricture was ascertained by history and radiographic imaging. We performed 36 Allium® ureteral stent placement procedures (32 patients) between May 2019 and June 2020. All procedures were conducted or supervised by a single consultant endourologist. All stent placement procedures stuck with the same technique as detailed below. The primary endpoint was the patency rate of the Allium® ureteral stent, while the secondary outcomes were the postoperative complications and GFR. Stent patency was defined as stent in situ without evident migration, unanticipated stent exchange or recurrent ureteral obstruction (increasing hydronephrosis and associated deterioration of renal function). The volume of hydronephrosis was evaluated according to the meridian measured by computed tomography (CT): hydronephrosis volume = length * width * depth * 0.523 [8, 9]. The GFR was evaluated by Single-Photon Emission Computed Tomography (SPECT). Postoperative complications were graded based on the Clavien-Dindo classification [10]. Cost analysis was calculated from stent cost, anesthesia cost and operating room fee.

### Technique

**Fig. 1:**
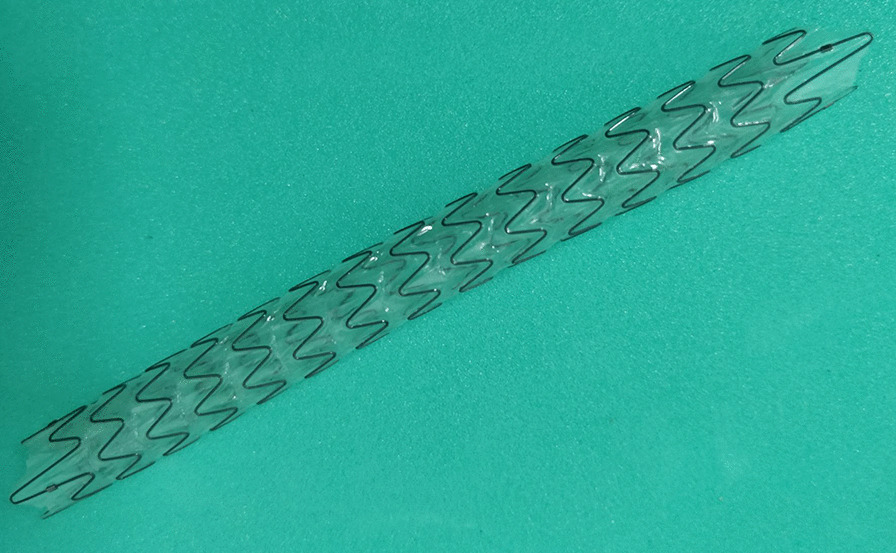
The Allium ureteral stent

The Allium ureteral stent has two calibers (24F and 30F) and two lengths (10 cm and 12 cm). The main body has a high radial force with softer end segments (Fig. 1). In all patients, the procedure was conducted in the lithotomy position under general anesthesia with a prophylactic antibiotic used. After rigid cystoscopy, a hydrophilic guidewire was inserted under fluoroscopic guidance to the renal pelvis. For patients who were not possible to pass a guidewire, a small channel was incised with holmium laser (1.1 to 1.3 j, 30 Hz) under the fluoroscopy and ureteroscope guidance. The ureters were contrasted anterogradely through the nephrostomy tube or retrogradely via ureteroscope under X-ray guidance to show the location and length of ureteral stenosis. Subsequently, a guide wire was introduced and a ureteral balloon dilator (21 F) was utilized to pass over the site of stenosis and inflated to 25 atm (2.53 MPa) for 3 min. Afterwards, Allium stent (10 or 12 cm, 24F or 30F) was introduced into the stricture segment and identified by fluoroscopic image. The operative time was the interval between insertion of cystoscope and the completion of stent placement. Length of hospital stay was defined as the duration passed from the operative day to the hospital discharge day.

### Follow-up

Kidney, ureter, bladder (KUB) X-ray was conducted 1 day after stent insertion to determine the position of the stent. CT imaging and SPECT were conducted in all patients 1 month after stent insertion. Then CT scan, creatinine measurement, urine cultures, urinalysis and serum biochemistry were carried out every 3 months in the stable patients.

### Statistical analysis

Continuous variables were presented with mean ± SD (standard deviation) or median (range) based on normal or non-normal distribution. Categorical variables were summarized with a count and percentage. A paired analysis (Student’s t-test or Wilcoxon signed rank test) was conducted with the volume of hydronephrosis, creatinine levels, blood urea nitrogen (BUN), GFR and hemoglobin (Hb) between preoperation and the last follow-up. SPSS v. 24 software (SPSS Inc. IBM Corp, Somers, NY, USA) were utilized to conduct statistical analysis. All tests were two-sided, and a *p* value of < 0.05 was deemed statistically significant.

## Results

From May 2019 until June 2020, we enrolled 32 patients (36 ureteral strictures) into the study. 11 patients suffered ureteral stricture following surgery and 6 patients suffered stricture following surgery and radiation therapy combined for cervical cancer.

Additionally, 4 patients suffered ureteral stricture following surgery and 6 patients suffered stricture following surgery and radiation therapy combined for rectal cancer. Five patients had stricture following surgery for ovarian cancer. The median length of ureteral stricture was 10.8 cm (range 3–17 cm) (Table 1).

**Table 1:** Patient demographics

Variable	US
Number of patients	32
Age, years, median (range)	46.8 (35–66)
Gender, male/female, n	7/25
BMI, kg/m^2^	22.9 ± 1.35
Etiology of stricture, n	
Surgery/radiation therapy for cervical cancer	17
Surgery/radiation therapy for rectal cancer	10
Surgery for ovarian cancer	5
Stricture side, n	
Unilateral	28
Bilateral	4
Stricture site, n	
Upper ureter	7
Middle ureter	4
Lower ureter	25
Length of US, cm, median (range)	10.8 (3–17)
Urinary infection before placement, n	5/32
Previous treatment, n	
Nephrostomy ≥ 1	2/32
PS ≥ 2	8/32
Endoincision ≥ 1	0/32
Balloon dilation ≥ 1	5/32

US, ureteral stricture; BMI, body mass index; PS, polymeric double-J stent

**Fig. 2:**
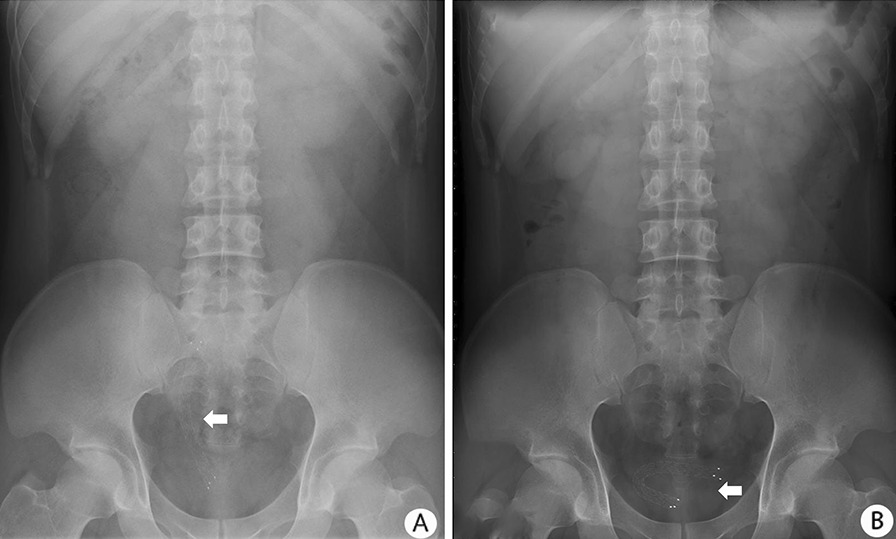
KUB radiography of stent displacement in patient with lower ureteral stricture 1 day and 6 months after insertion: **A** KUB radiography showing the normal position of stent 1 day after insertion; **B** KUB radiography demonstrating the stent migration into the bladder 6 months after placement

All stents were inserted successfully and positioned correctly in the 36 ureters. The procedure-related characteristics were demonstrated in Table 2. The total cost of per patient in our study was $10,600.2 US dollars (range $9394.4–$33,527.4 US dollars). During a median follow-up time of 16 months (range 8–21 months), 27 cases (31 sides, 84%) remained stent patency. Twelve patients died from their primary malignancy carrying a patency stent. Three stents occluded and 4 stents migrated in our study. Two stents occluded due to the obstruction of urinary blood clots. Three (9.3%) and four (12.5%) patients had postoperative fever and gross hematuria, respectively. Two patients (6.2%) suffered the urinary tract infection postoperatively. The patients were managed by antibiotics treatment. Stent migration (3, 4, 6, and 10 months after the procedure) occurred in 4 (12.5%) ureters (one into the bladder, two into renal pelvis, and one within ureter). Primary stricture site of ectopic stents includes 2 cases of upper, 1 case of middle and 1 case of the lower ureter. The median length of ureteral stricture of 4 patients who suffered stent migration was 5.4 cm (range 3.9–7.9 cm). As shown in Fig. 2, the stent migrated into the bladder 6 months after stent placement. Ectopic stents were endoscopically removed and a new Allium® ureteral stent was reinserted successfully. In all four patients, no clinical symptoms of fever or infections were observed, and computed tomography revealed no aggravated hydronephrosis. There were no patients experienced stent encrustation in our study (Table 2).

**Table 2:** Procedure-related characteristics and follow-up

Variable	US (n = 32)
Operative time, min, median (range)	75 (40–125)
Length of hospital stay, days, median (range)	7 (4–17)
Total cost, $, median (range)	10,600.2 (9394.4–33,527.4)
Stent cost, $, median (range)	7558 (7377–7992)
Anesthesia cost, $	270
Operating room fees, $	2200
Follow up, months, median (range)	16 (8–21)
Stent patency, n (%)	27 (84)
Postoperative complications, n (%)	
Clavien Dindo grade I	
Gross hematuria	4 (12.5)
Fever	3 (9.3)
Clavien Dindo grade II	
Infection needed antibiotics	2 (6.2)
Clavien Dindo grade III–IV	
Stent migration	4 (12.5)
Stent encrustation	0

The volume of hydronephrosis postoperatively was significantly reduced compared with preoperation (54.18 ± 15.42 vs 23.92 ± 8.3, *P* = 0.019). There was no statistically significant difference regarding GFR (22.53 ± 6.55 vs 25.1 ± 13.1, *P* = 0.33), creatinine levels (103.45 ± 31.86 vs 90.1 ± 30.96, *P* = 0.082), BUN (6.77 ± 1.98 vs 5.76 ± 1.57, 0.134) and Hb (113.2 ± 28.5 vs 117.8 ± 22.9, *P* = 0.14) between preoperation and last follow-up (Table 3).

**Table 3:** Difference between preoperation and last follow-up

Factors	Preoperation	Last follow-up	*p* value
Volume of hydronephrosis/cm^3^	54.18 ± 15.42	23.92 ± 8.3	0.019
Creatinine levels/(µmol/L)	103.45 ± 31.86	90.1 ± 30.96	0.082
BUN/(µmol/L)	6.77 ± 1.98	5.76 ± 1.57	0.134
Norm GFR of affected kidney/(mL/min/1.73 m^2^)	22.53 ± 6.55	25.1 ± 13.1	0.33
Uptake of affected kidney (%)	30.9 ± 6.26	34.3 ± 8.69	0.161
Hb/(g/L)	113.2 ± 28.5	117.8 ± 22.9	0.14

BUN, blood urea nitrogen; GFR, glomerular filtration rate; Hb, hemoglobin

## Discussion

This is the largest study evaluating the efficacy and safety of Allium ureteral stent in the management of ureteral stricture following surgery and/or radiation therapy for malignancy. The current study showed that Allium ureteral stent patency rate was 84% during a median observation time of 16 months. Twelve patients died from their primary malignancy carrying a patency stent. The placement of Allium stent significantly decreased average volume of hydronephrosis from preoperative 54.18 cm^3^ to postoperative 23.92 cm^3^ (*P* = 0.019). Three stents occluded and 4 cases of stent migrated. No encrustation was observed in our series. Three (9.3%) and four (12.5%) patients had postoperative fever and gross hematuria, respectively. Infection was observed in 2 cases (6.2%), mandating antibiotics therapy. Additionally, the total cost of per patient in our study was $10,600.2 US dollars (range $9394.4–$33,527.4 US dollars). Therefore, our study demonstrated that Allium ureteral stent is effective and safe in the treatment of ureteral stricture following surgery and/or radiation therapy for malignant cancer.

It is reported that nearly 75% of all ureteral strictures were caused by iatrogenic injury and radiation treatment [1]. Ureter is prone to be injured during gynecologic and general surgery as it is proximal to anatomical position of ovaries, cervix, colon and rectum [11]. In addition, the delicate blood supply of ureter renders it less likely to self-repair after injury. Common mechanisms of iatrogenic surgery are direct injury (crushing, squeezing, transection, suture ligation and coagulation) and indirect injury (devascularization, energy induced and generation of free oxygen radicals) [12]. Iatrogenic ureteral stricture is more likely to be induced in the lower third of ureter than the middle and upper third [13, 14]. Nearly 70% of stricture located in the lower third ureter in our study, which is consistent with the previous report [13, 14].

The Allium ureteral stent is a fully covered, self-expanding, large caliber metal stent. The stent is made of super elastic nickel-titanium alloy, which maintain lumen patency via providing long-term direct wall support. The stent patency rate was 84% during a median observation time of 16 months in our study, which is slightly lower compared with previous study [15]. Zhong et al. reported a success rate of 87.5% with the use of Allium ureteral stent in the treatment of ureteral stricture after kidney transplantation [15]. However, the mean length of ureteral stricture was 2.5 cm in their series. Moskovitz et al. reported that a total success rate of 95% with the insertion of Allium ureteral stent for a median indwelling time of 17 months [6]. The patients who underwent stent exchange and had a secondary luminal patency were also considered as successful [6]. In contrast, unanticipated stent exchange was regarded as stent failure in our study. The distinct success definition might account for our slightly lower stent patency rate.

The covered biocompatible, biostable polymer makes Allium ureteral stent a nonpermeable tube to avoid tissue ingrowth and early encrustation. In our study, no cases of stent encrustation were observed, which might be attributed to the specific unraveling materials of stents. Similarly, previous study published by Moskovitz and colleagues did not observe encrustation with the placement of Allium ureteral stent [6]. Stent migration is a relatively rare but significant postoperative complication. Polymer double-J stent provides self-retaining capability because of J-shaped design at two extremities which anchor the stent in the renal pelvis and the bladder. Compared with traditional double-J stent, Allium stent has a large caliber and strong supporting force. In our series, stent migration occurred in 4 out of 36 (12.5%) placements, which is slightly lower than the previous report of 14.2% [6]. In two patients, the stent migrated into the renal pelvis, while the other two stents migrated into bladder and within ureter, respectively. Primary stricture site of ectopic stents includes 2 cases of upper, 1 case of middle and 1 case of the lower ureter. Three patients with stent migration had engaged in bending, lifting heavy objects and running. Consequently, we speculated that the displacement of stents might be related to ureteral peristalsis and physical activity of patients. Ectopic stents were endoscopically removed and a new Allium ureteral stent was reinserted successfully. In all four patients, no clinical symptoms of fever or infections were observed, and CT scan revealed no aggravated hydronephrosis.

The volume of hydronephrosis is significantly decreased from preoperative 54.18 cm^3^ to postoperative 23.92 cm^3^ after stent insertion (*P* = 0.019), demonstrating that the Allium stent was effective for relieving hydronephrosis and function protection. Similarly, Dong et al. reported that the average width of the renal pelvis significantly reduced from 2.65 to 1.34 cm after stent placement procedure [16], which supports our results. The stent patency rate was 84% during a median observation time of 16 months in our analysis, and the cost of Allium stent was based on the annual procedure. Cost for single Allium ureteral stent is between $7377 to $7992 US dollars, and the anesthesia and operating room fee were $270 and $2200 US dollars. Therefore, the median total cost was $10,600.2 US dollars (range $9394.4–$33,527.4 US dollars) in our analysis. In contrast, annual cost for double-J stents was extrapolated on the basis of stent exchange made every 3 to 6 months. The cost for single polymer stent insertion is between $2255 and $3125 US dollars, and the extrapolated annual cost for polymer stent would then be $9648 to $13,128 US dollars [17], which is comparable to the annual cost for Allium stent. The annual charge for Resonance metal stent was $9469.5 US dollars [18], this analysis was conducted based on Resonance stent exchange only once a year. Additionally, the annual cost for another thermo-expandable nickel-titanium alloy ureteral stent, Memokath 051, was calculated at $7841 US dollars [19], which is slightly lower than Allium stent. However, 17% of re-operation rate for Memokath 051 stent migration and/or occlusion might negate the cost savings potential in patients [19]. Two patients had an average total cost more than $30,000 US dollars. Both of them had bilateral ureteral stricture and two Allium stents were inserted during the procedure. Our results demonstrated a stent median life surpassing 1 year in the majority of patients. Longer stent life results in less frequent stent exchange, decreased patients’ discomfort and improved quality of life.

Polymeric double-J stent is an appealing first choice for managing ureteral stricture. Moreover, no special facilities are required and all urologists are familiar with this common procedure. However, application of polymeric stent can be plagued with an approximately 20% failure rate [20] and complications such as urinary tract infection, encrustation and obstruction [21]. The Resonance metallic stent has a tightly coiled wire structure and is made of Ni-Co-Cr-M alloy. Several retrospective studies have been conducted with a 65–78% stent patency rates at various duration of follow-up [22–24]. Notably, the effectiveness of Resonance in alleviating ureteral obstruction was not observed in children, and pediatric patients needed further treatment to relieve the obstruction [25]. In addition, a number of studies have reported the efficacy of Memokath 051 metal-mesh ureteral stent. Agrawal et al. prospectively collected data on patients who had a Memokath 051 stent insertion [26]. 14 patients (19%) needed re-insertion during a mean follow-up of 7.1 months [26]. They also identified chronic kidney disease, diabetes and ureteropelvic junction strictures as predictive factors for migration and the need for re-insertion. Another study published by Maan et al. evaluated stent-related symptoms between double-J stent and the Memokath 051 [27]. They found that the Memokath group significantly outperformed the double-J stent group regarding general health, pain and urinary symptom index. However, this conclusion should be drawn cautiously because of retrospective biases of study. In addition to single ureteral stent insertion, it is possible to place 2 stents side-by-side (Tandem ureteral stents). Tandem ureteral stents improve the urine flow through providing additional scaffolding to withstand the compressive forces. In addition, the use of 2 ipsilateral stents increases the stiffness and reduces kinking [28]. Vogt el al. switched to tandem stents after obstruction of the stiffest stent in less than 6 months [29]. They performed 18 tandem ureteral stent insertion and found 2 cases (11.1%) experienced stent failure, which might be predictive for a decrease in survival [29, 30]. Active clinical and ultrasound are also needed in patients with tandem stents to evaluate renal function.

The current study had several limitations. Firstly, we could not compare the efficacy and safety between Allium ureteral stent and polymer double-J stent. Secondly, stent-related symptoms were not assessed by questionnaire in the current study. However, this is the largest study evaluating treatment outcomes of Allium ureteral stent in the ureteral stricture following surgery and/or radiation therapy for malignancy. We also performed the cost analysis of Allium ureteral stent in the management of stricture for the first time.

## Conclusions

In summary, the current study demonstrated that Allium ureteral stent is effective and safe for patients with stricture following surgery and/or radiation therapy for malignancy. The stent could maintain a high patency rate and low complication rate for a long period of time. Moreover, patients’ hydronephrosis could be improved by the stent placement.

## Data Availability

The datasets used and or analyzed during the current study available from the corresponding author on reasonable request.

## Abbreviations

KUB: Kidney, ureter, bladder
SD: Standard deviation
BUN: Blood urea nitrogen
GFR: Glomerular filtration rate
Hb: Hemoglobin
